# Transparent Polyurethane Nanofiber Air Filter for High-Efficiency PM2.5 Capture

**DOI:** 10.1186/s11671-019-3199-0

**Published:** 2019-12-02

**Authors:** Wen Liang, Yuan Xu, Xiao Li, Xiao-Xiong Wang, Hong-Di Zhang, Miao Yu, Seeram Ramakrishna, Yun-Ze Long

**Affiliations:** 1 Collaborative Innovation Center for Nanomaterials & Devices, College of Physics, Qingdao University, Qingdao, 266071 China; 2 Qingdao Junada Technology Co., Ltd, Qingdao International Academician Park, Qingdao, 266199 China; 3 Department of Mechanical Engineering, Columbia University, New York, NY 10027 USA; 4 Center for Nanofibers & Nanotechnology, Faculty of Engineering, National University of Singapore, Singapore, Singapore; 5 Collaborative Innovation Center for Eco-Textiles of Shandong Province, Qingdao University, Qingdao, 266071 China

**Keywords:** Electrospinning, PM2.5, Nanofibers, Air filter, Removal efficiency

## Abstract

Fine particulate matter (PM) has seriously affected human life, such as affecting human health, climate, and ecological environment. Recently, many researchers use electrospinning to prepare nanofiber air filters for effective removal of fine particle matter. However, electrospinning of the polymer fibers onto the window screen uniformly is only achieved in the laboratory, and the realization of industrialization is still very challenging. Here, we report an electrospinning method using a rotating bead spinneret for large-scale electrospinning of thermoplastic polyurethane (TPU) onto conductive mesh with high productivity of 1000 m^2^/day. By changing the concentration of TPU in the polymer solution, PM2.5 removal efficiency of nanofiber-based air filter can be up to 99.654% with good optical transparency of 60%, and the contact angle and the ventilation rate of the nanofiber-based air filter is 128.5° and 3480 mm/s, respectively. After 10 times of filtration, the removal efficiency is only reduced by 1.6%. This transparent air filter based on TPU nanofibers has excellent filtration efficiency and ventilation rate, which can effectively ensure indoor air quality of the residential buildings.

## Introduction

Fine particulate matter (PM) is composed of various solid fine particles and droplets with up to hundreds of chemical components. PM is mainly composed of three major chemical substances, including water-soluble ions, carbon-containing compounds, and other inorganic compounds [1–5]. PM is mainly from the burning of fossil fuels and garbages, and it is rich in toxic substances and harmful particulate matter [1, 3–6]. According to the size of the particle diameter, PM is mainly divided into PM2.5 and PM10, which means that the aerodynamic diameter of the particles is less than 2.5 μm and 10 μm. PM10 stays in the air from a few minutes to a few hours with a limited travel distance; however, PM2.5 has a long residence time in the atmosphere and can last from several days to several weeks [2, 5]. Even if PM2.5 falls to the ground, it is easy to be blown back into the air by the wind. Through the process of breathing, PM2.5 can enter the body and accumulate in the trachea or the lung, which will negatively affect the human health [7–9]. PM2.5 also has a major impact on the climate and the ecological environment, such as affecting the rainfall process [10–14]. In the past 10 years, PM2.5 air pollution is becoming more and more serious, especially in some developing countries such as China and India [4, 15]. In daily life, people at those countries often encounter severe haze weather. For this reason, it is very necessary to take some protection against PM2.5.

At present, the protection measures to the severe haze are mainly focused on the outdoor personal protection, such as wearing professional dust masks, which can effectively filter the particle matter [16, 17]. The indoor personal protection, such as ventilation systems and air purifier are expensive, complicated to install and requiring replacement for the filter elements [6]. The indoor air filters generally provide air protection for commercial building, due to the high cost of pumping systems for active air exchange. Recently, there are two transparent air filters for residential buildings by windows passive ventilation come into the vision of consumer [17]. One is porous membrane filter, but the porosity of this filter is very low, which means high ventilation cannot be achieved. Another one is nanofiber air filter, which porosity can reach 70% and can achieve high ventilation. Some laboratories have prepared a variety of window screens to protect the quality of indoor air with nanofiber. For instance, Chen et al. [18] reported an air filter prepared using electrospun TPU polymer; TPU nanofiber air filter is very effective for removing PM2.5 (98.92%) with very low-pressure drop (10 Pa). Khalid et al. [19] reported a nanofiber window screen made by direct blowing technology, which has good optical transparency (80%) and high PM2.5 filtration efficiency (99%). Liu et al. [6] prepared a transparent air filter by electrospinning, which achieved high ventilation and high PM2.5 filtration efficiency (> 95.0%). However, this research was developed in laboratories and the research of the industrial process of nanofiber filter is little.

In recent years, electrospinning technology has received extensive attention due to its low energy consumption, simple operation, and environmentally friendly methods for preparing nanofibers [20, 21]. Nanofiber membranes prepared by electrospinning has high porosity, micro-nano channel interconnects, and high specific surface area [22–29]. Recently, our team developed a TPU nanofiber air filter that can be mass-produced using a spinning bead spinneret [30, 31]. This air filter has very high thermal stability, good optical transparency of 60%, high PM2.5 removal efficiency of 99.654%, long lifetime, low airflow resistance (ventilation rate 3348 mm/s), and light weight.

## Experimental

### Materials and Instruments

Polymer TPU was obtained from Bayer Co., Ltd., Germany, with tear resistance, abrasion resistance, and UV protection; the substrate conductive mesh is provided by Qingdao Junada Technology Co., Ltd., China. The *N,N*-dimethylfomamide (DMF) and acetone were provided by the Tianjin Zhonghe Shengtai Chemical Co., Ltd. Scanning electron microscopy (SEM Feiner High Resolution Professional Edition Phenom Pro) is used to study the morphology of TPU fibers. An automatic filtration performance tester for evaluating filtration performance FX3300 Lab Air-IV was purchased from Shanghai Lippo Co., Ltd., China. AFC-131 is used to test ventilation rate purchased from Shanghai Huifen Electronic Technology Co., Ltd. Thermo Scientific Nicolet iS5 is used to measure infrared and analyze the functional groups of TPU fiber membranes. Theta optical contact angle meter was used to analyze the contact angle of TPU fiber film. The light transmittance was evaluated using a UV1901PC ultraviolet spectrophotometer and purchased from Shanghai Aoxiang Scientific Instrument Co., Ltd., China.

### Preparation of Nanofibrous Membranes

**Fig. 1:**
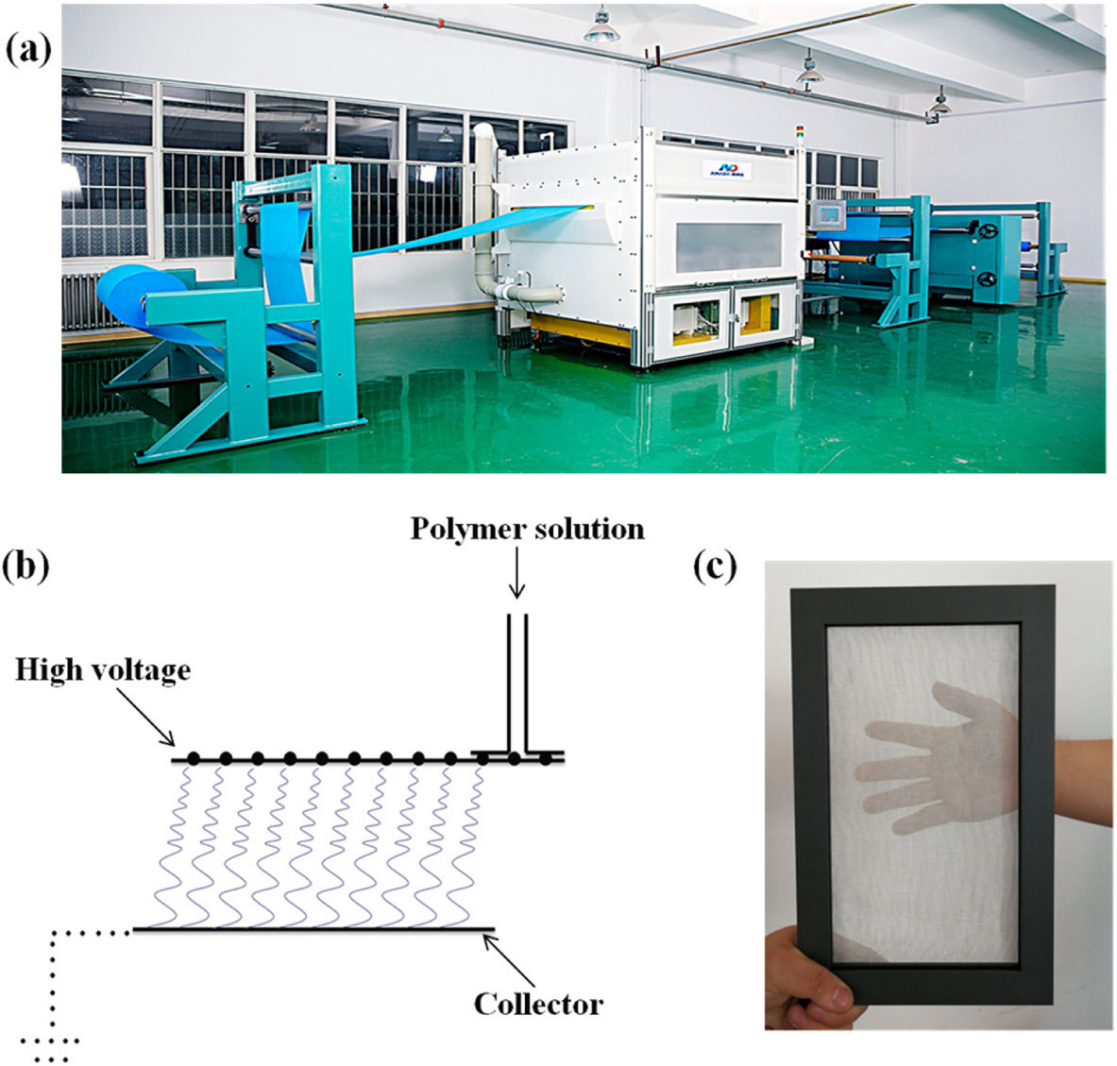
Electrospinning equipment. **a** A picture of the electrospinning apparatus used in this work. **b** Schematic diagram of the electrospinning setup with rotary bead-wire spinnerets. **c** The nanofiber membrane in this air filter is a sample produced by the electrospinning apparatus

TPU nanofiber membrane was fabricated using electrospinning equipment NES-1 (Qingdao Junada Technology Co., Ltd.), which is displayed in Fig. 1a. The mainframe is 2350 mm long, 2200 mm wide, 2700 mm high, and weighs 1980 kg. The touch screen is Siemens PLC, the power is 30 kV, and the spinning width is 1.1 m. The average fiber diameter is about 120 nm, and the weight of the nanofiber membrane is about 0.5 g per square meter. The substrate is suitable for cellulose, synthetic fiber, etc., and the polymer material is suitable for TPU, PVP, PAN, etc. The electrospinning principle is shown in Fig. 1b, and schematic diagram of a nanofiber membrane produced by electrospinning is shown in Fig. 1c. The solution used in the electrospinning was to dissolve different masses of TPU in a mixed solvent in a ratio of DMF to acetone in a volume ratio of 1:1; the spinning voltage was positive pressure 30 kV and negative high pressure − 30 kV, which resulted in a stable jet; substrate moving speed was 10 m/min; and the spinning distance was controlled at 200 mm. The temperature and relative humidity during this process were controlled at 25 °C and 50% RH. In order to get different average diameters of nanofibers, the concentration of TPU in the solution was adjusted from 6 to 16 wt%. The TPU solution was electrospun onto conductive mesh under the same conditions. The different concentrations of TPU fiber membranes were named TPU-6, TPU-8, TPU-10, TPU-12, TPU-14, and TPU-16, respectively.

## Results and Discussion

### Characterization of Morphology and Structures

**Fig. 2:**
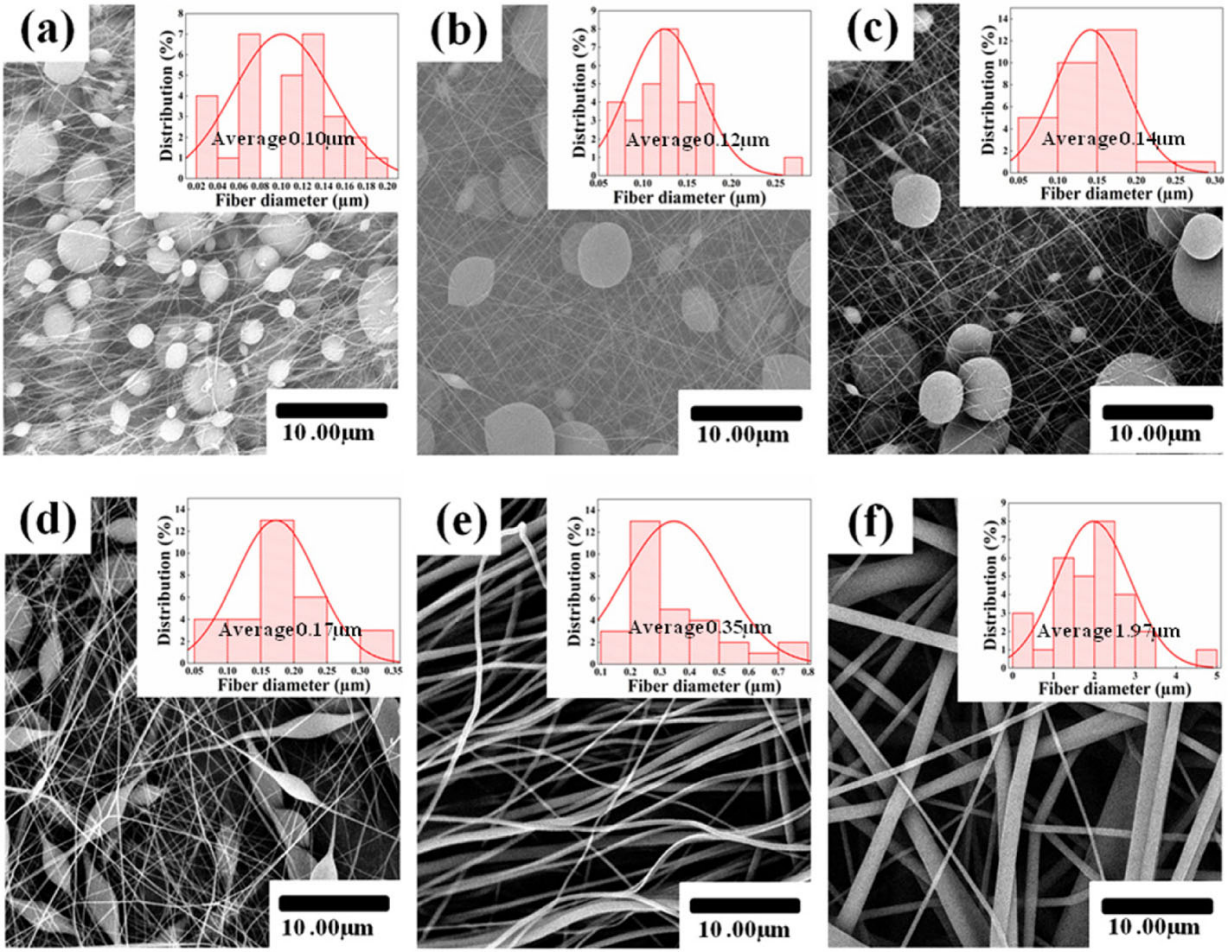
SEM images of electrospun TPU. a–f SEM images and diameter distributions with TPU concentrations of 6 wt%, 8 wt%, 10 wt%, 12 wt%, 14 wt%, and 16 wt%, respectively

One of the important trends in the membrane characterization of nanofibers is the morphology of the membrane surface. The morphology of the TPU nanofiber membrane was observed by SEM, and the voltage used was a 10 kV, scanning imaging system. As shown in Fig. 2a–f, the microscopic morphologies of the nanofiber membrane obtained from the electrospinning TPU solution are showed under different TPU concentrations of 6 wt%, 8 wt%, 10 wt%, 12 wt%, 14 wt%, and 16 wt%, respectively. When the TPU concentrations between 6 wt% and 12 wt% (Fig. 2a–d), there are many bead-like nanofibers of different sizes. This can be attributed to the low viscosity of the polymer TPU molecular chain with the low concentration of the TPU solution. Therefore, in the process of electrospinning low concentration TPU solution, the ejection was difficult to resist the stretching of the electric field force [32]. In addition, due to the viscoelasticity of the TPU molecular chain, the ejection stretched by the electric field force will aggregate to form beaded nanofibers [33]. However, as the concentration of TPU increases, the viscosity of the solution increases, and the electrospinning process will form nanofibers instead of beaded nanofibers, so the beaded nanofibers become less and less and eventually disappear completely (Fig. 2e–f). On the other hand, the viscosity of the solution is an important parameter affecting the diameter of the nanofiber [34]. When the concentration of the TPU solution increases, the viscosity of the solution also increases, so the diameter of the nanofiber increases, as shown in Fig. 2a–f. When the concentration of TPU is higher than 14 wt%, the diameter of nanofibers increases rapidly (Fig. 2e–f). The average diameter of the nanofiber is calculated by NanMeasurer. The average TPU nanofiber diameter is ~ 0.10 μm, ~ 0.12 μm, ~ 0.14 μm, ~ 0.17 μm, ~ 0.34 μm, and ~ 1.97 μm, corresponding to TPU-6, TPU-8, TPU-10, TPU-12, TPU-14, and TPU-16.

### Fourier Transform Infrared Spectrum Analysis

**Fig. 3:**
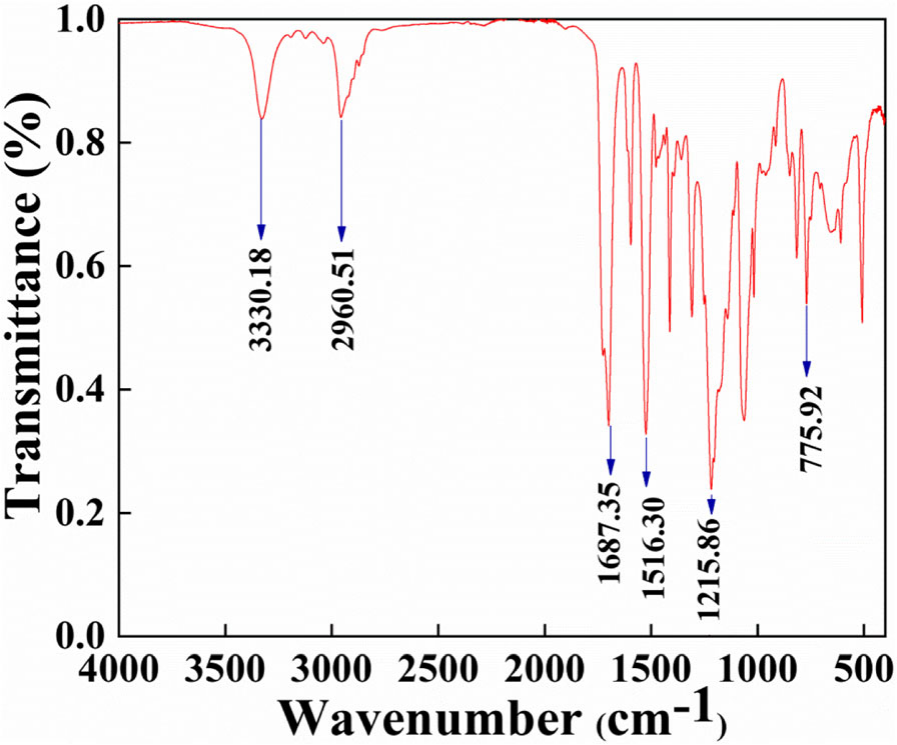
Composition of TPU nanofiber membrane. FTIR demonstration of TPU indicating the presence of various functional groups

To identify the composition of the prepared TPU nanofiber membrane, it is necessary to carry out Fourier transform infrared spectroscopy (FTIR) analysis on the sample. First, preheat the equipment for one and a half hours, the pressure is controlled at 15 Mpa, the working voltage is 220 V, the ambient temperature is controlled at 20 °C, the ambient humidity is controlled at 40%, the frequency is 50 Hz, and the current is 7.5 A. The test results are as shown in Fig. 3, which is obviously the same as the infrared spectrum of the substrate polyurethane. The spectrum is shown in Fig. 3. Strong absorption peaks were observed at wave numbers 3330.18 cm^-1^, 2960.51 cm^-1^, and 1215.86 cm^-1^, indicating the presence of N–H and C–H functional groups. The surface of the TPU nanofiber has hydrophobic functional groups, and the surface of the fiber membrane is smooth and dense. So, the prepared transparent air filter has a certain hydrophobic function. Due to the hydrophobic nature of the TPU fiber membrane, the TPU transparent air filter can open the window on rainy days.

### Filtration Efficiency Analysis

**Fig. 4:**
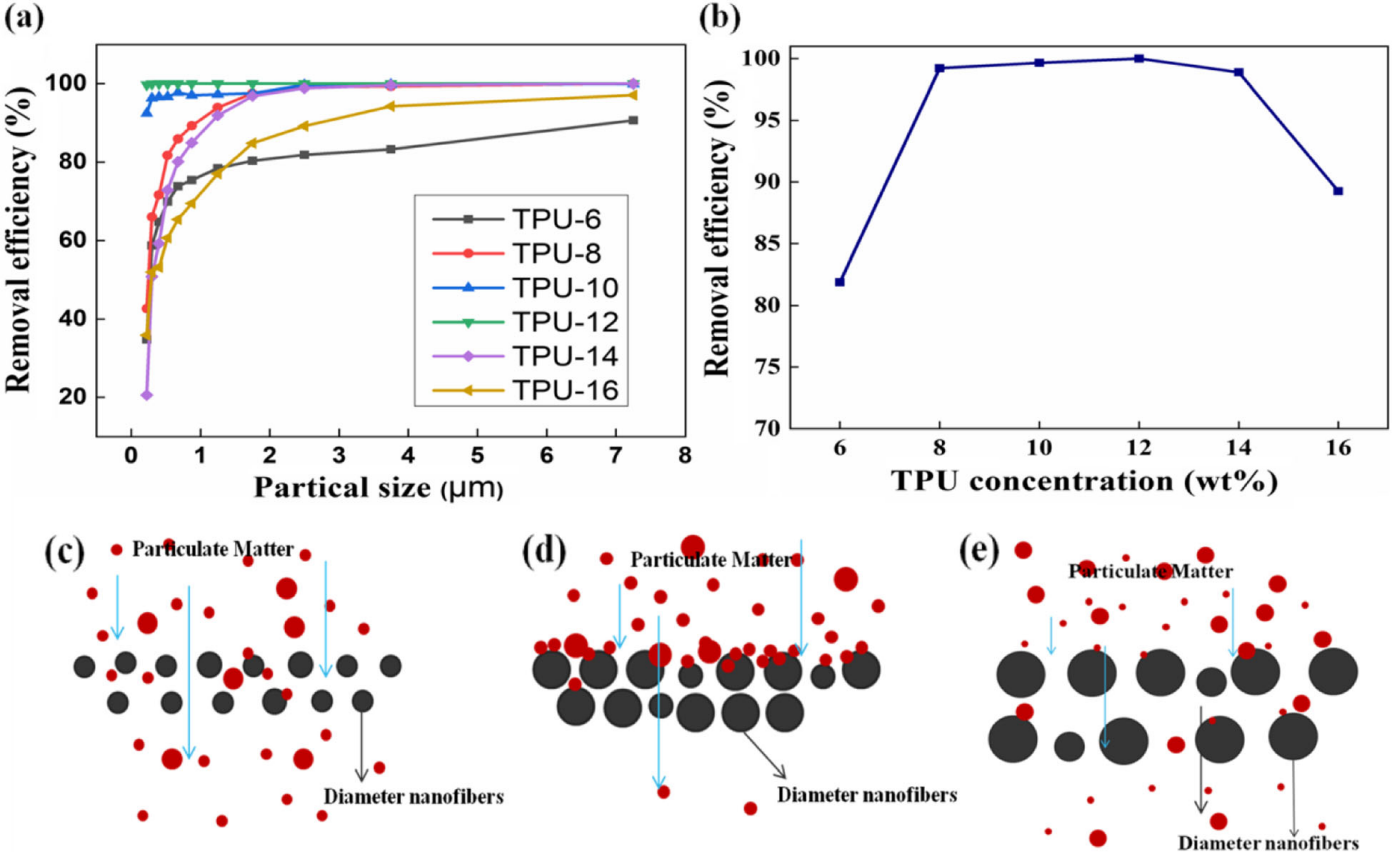
Evaluation of filtration efficiency of TPU fiber membrane. **a** Remove efficiency of PM of different sizes with TPU concentrations of 6 wt%, 8 wt%, 10 wt%, 12 wt%, 14 wt%, and 16 wt%, respectively. **b** PM2.5 removal efficiency of different concentrations of TPU fiber membranes. **c**–**e** PM through different diameter fiber membranes

Filtration efficiency is the most important parameters for evaluating transparent air filters. The filtration efficiency test was carried out on different TPU fiber membranes. In this study, the test conditions were the same, the temperature was 20 °C, the relative humidity was 40.6%, the flow rate is 2.0 m^3^/h, and PM pollutants are aerosol particles. The size distribution of PM and the filtration effect of each sample are shown in Fig. 4a. The filtration efficiency is positively correlated with the PM particle size. For the same size of PM particles, such as PM2.5 (Fig. 4b), with the TPU concentration increases from 6 to 12 wt%, the removal efficiency is significantly increased, which can be attributed to the fact that the membrane waved by nanofibers with larger diameter are better to resistant PM particles. However, with the TPU concentration increases from 12 to 16 wt%, the increase in the spacing between the fibers and the disappearance of the bead string fibers results in a significant decrease in the removal efficiency of the TPU fiber membrane [18]. The increase in the concentration of the solution makes the elongation of the electrospinning jet more difficult and slower, resulting in an increase in the pore size of the TPU fiber membrane. Figure 4c–e shows the passage of particulate matter through different diameter fiber membranes. The larger fiber diameter effectively prevents the PM from passing through the fiber membrane, and as the TPU concentration becomes larger, the fiber diameter becomes larger, but the distance between the phase fibers also becomes larger, resulting in a decrease in filtration efficiency. The highest removal efficiency of PM2.5 is the TPU-12. When the particle diameter is **≥** 0.525 μm, the removing efficiency is 100%, and the pressure drop is only 10 Pa. In addition, the TPU-10 on PM2.5 removing efficiency is 99.654%.

### Ventilation Rate Analysis

**Fig. 5:**
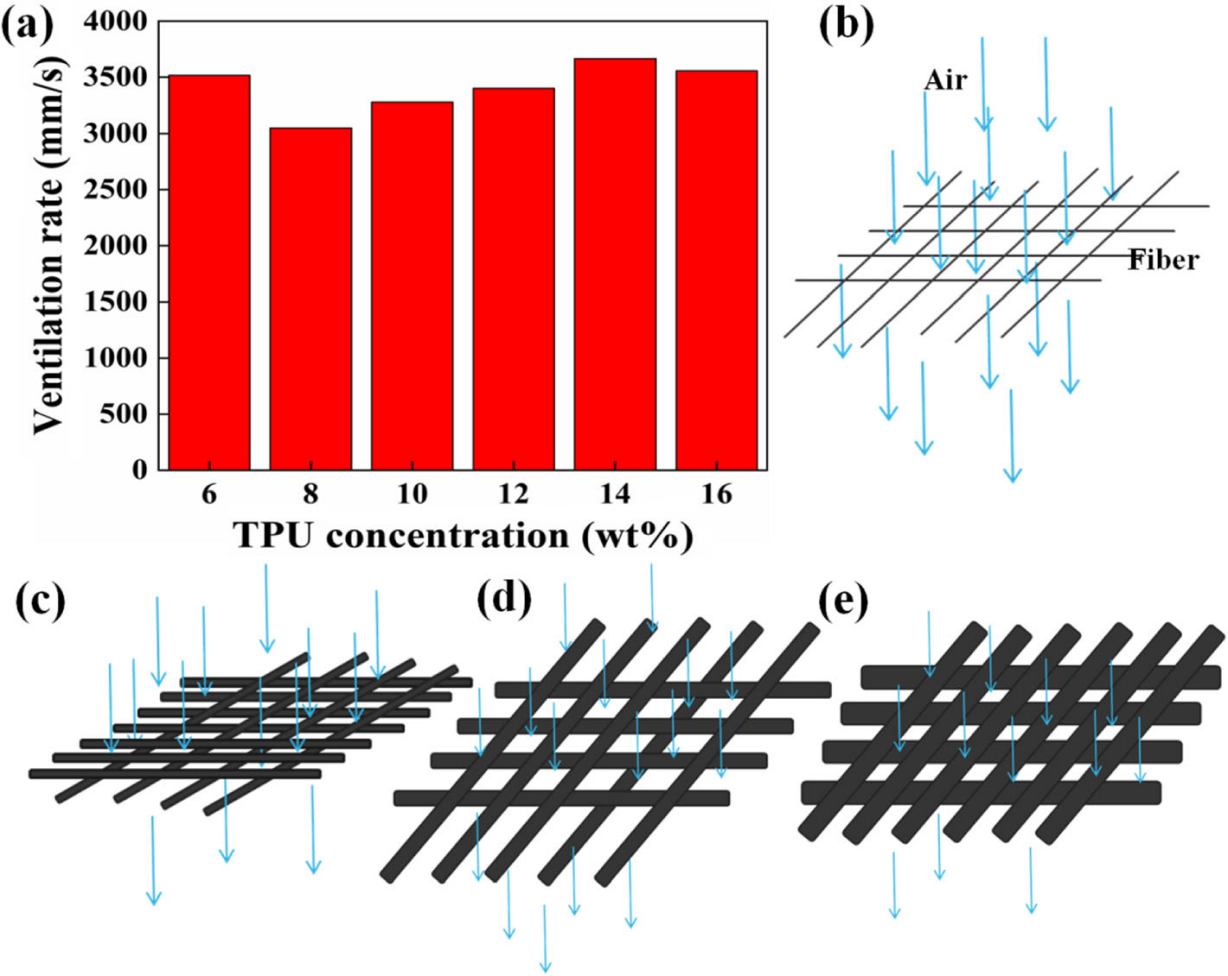
Ventilation rate evaluation of TPU fiber membrane. **a** Ventilation rate of different concentrations of TPU fiber membranes. **b**–**e** Air passes through fibers of different diameters

Maintaining high ventilation is an important property to evaluate the performance of the air filter. Six samples were tested for ventilation rate under the same conditions. The measurement area was 20 cm^2^ and the measurement pressure was 200 Pa. The ventilation rate of different concentrations of TPU nanofiber membranes is shown in Fig. 5a, and the corresponding pressure drop is 6 Pa, 15 Pa, 12 Pa, 10 Pa, 7 Pa, and 9 Pa. The ventilation rate of different TPU membranes begin falling first, then maintains increases and lastly falling slightly, corresponding to the solution concentration increasing from 6 to 8 wt%, 8 to 14 wt%, and 14 to 16 wt%. There are two main reasons for affecting the ventilation rate: nanofiber packing density and the fiber average diameter [34]. The nanofiber packing density is calculated as follows:

1 $$ \alpha =\frac{W}{\rho_fZ} $$

Here, *α* is the nanofiber packing density, *W* is the basis weight of the nanofiber membrane, *ρ*_*f*_ is the density of nanomaterial, and *Z* is the nanofiber film thickness. The ventilation rate begins to decline is primarily owing to the addition of TPU nanofiber average diameters (Fig. 5b, c). As the concentration of TPU increases from 8 to 14 wt%, decreasing in the packing density of nanofibers leads to an increase in the distance between the nanofibers, which is beneficial to ventilation rate, even though the diameter of the nanofibers is increased (Fig. 5d). When the nanofiber membrane is made of a solution with a TPU concentration of 14 to 16 wt%, nanofiber diameter plays a crucial role in ventilation rate, and the associated ventilation rate drops slightly (Fig. 5e). When the TPU concentration increases to 10 wt%, the ventilation rate is up to 3480 mm/s, such a high ventilation rate is equivalent to a blank screen without a nanofiber membrane.

### Contact Angle Analysis

**Fig. 6:**
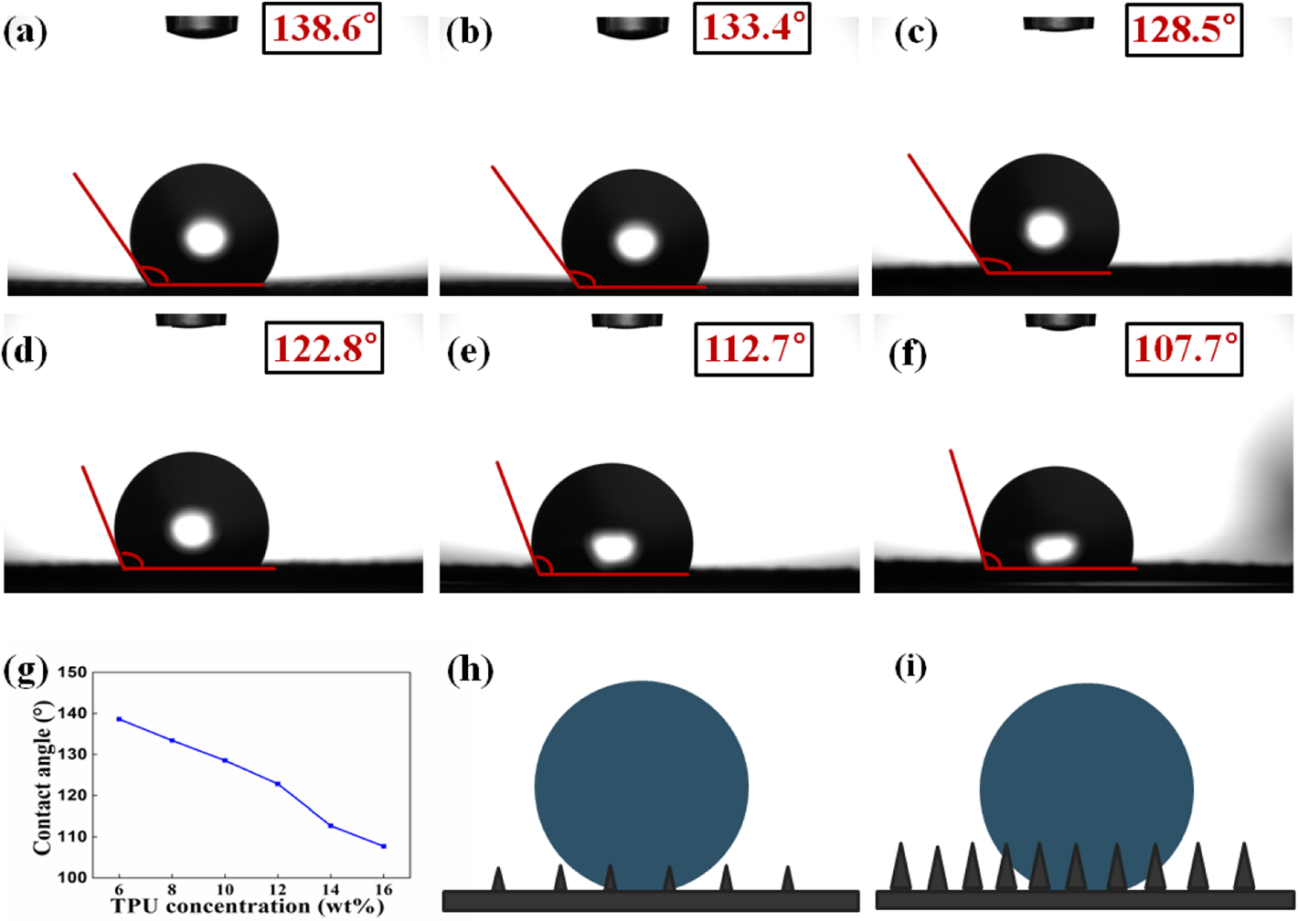
TPU fiber membrane contact angle characterization. a–f Testing the contact angle of different concentrations of TPU fiber membranes using 5-μL droplets. **g** Contact angle of different concentrations of TPU fiber membrane. **h**–**i** Droplets on the surface of fibers of different diameters.

Hydrophobicity is an important parameter for evaluating the performance of air filters, and the wettability of obtained TPU fiber membrane was measured by DSA using a 5-μL droplet. The results are shown in Fig. 6a–f, the contact angles are 138.6°, 133.4°, 128.5°, 122.8°, 112.7°, and 107.7°, corresponding to TPU-6, TPU-8, TPU-10, TPU-12, TPU-14, and TPU-16. The contact angle of all samples was greater than 90°, indicating that the transparent air filter prepared with polymer TPU is highly hydrophobic due to the hydrophobic functional groups on the surface of the TPU nanofiber membrane, the small fiber diameter leads to smooth membrane surface and fiber membrane dense structure. However, as the concentration of TPU becomes larger, the contact angle becomes lower and lower (Fig. 6g), because the roughness of the surface of the fiber membrane becomes larger. The relationship between contact angle and surface roughness of nanofiber membrane can be understood by Wenzel equation, which is defined as follows:

2 $$ \cos \theta '=r\cos \theta $$

Here, *r* is the surface roughness factor, which is the proportion of the actual area of the surface to the geometric projected area ( *r* ≥ 1), *θ*^′^ is the contact angle of the rough surface. As shown in Fig. 6h–i, with the TPU concentration increases, the diameter of the TPU nanofiber increases, and increased roughness of the surface of the nanofiber membrane, resulting in an increasingly low contact angle.

### Transparency and Reproducibility Testing

**Fig. 7:**
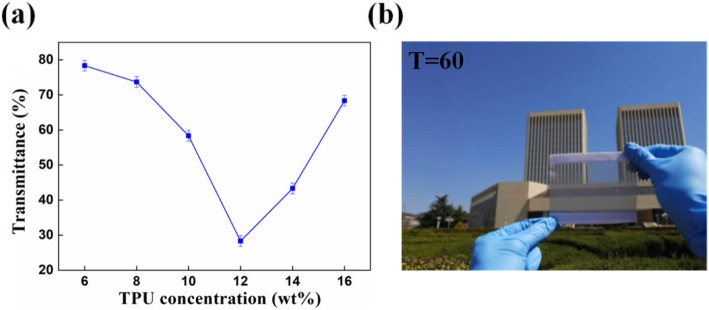
Transmission properties of TPU fiber membrane. **a** Transmittance of different concentrations of TPU fiber membrane. **b** Photographs of TPU concentration of 10 wt% transparent air filters at 60% transparency

Another important parameter of the transparent air filter is transmission; the transmittance of the six samples was tested and the results are shown in Fig. 7a. It was found that the transmittance first kept decreasing and then increased, corresponding to the increase in TPU concentration from 6 to 12 wt% and 12 to 16 wt%. When the TPU concentration is from 6 to 12 wt%, the transmittance of the fiber membrane is gradually reduced, mainly because the solution concentration is too low at the beginning (such as 6 wt% and 8 wt%), and the electrospinning process does not easily form fibers. When the concentration of the solution increases, the solution concentration is more suitable for electrospinning, so that more and more fibers are formed by electrospinning. The nanofiber diameter also becomes larger, and the fiber membrane becomes thicker and thicker, so that less light can pass through the fiber membrane. On the other hand, since the concentration of the solution is too low, electrospinning forms a large number of beads (Fig. 2a–d), which is adverse for light to pass through the fiber membrane. When the solution concentration is from 12 to 16 wt%, the transmittance of the fiber membrane gradually increases, mainly because the viscosity of the solution increases, and the electrospinning process becomes difficult gradually, so that less nanofiber is produced. Another reason is that as the concentration of the solution increases, the beaded string disappears, contributing more light to pass through the fiber membrane. Transmittances of 80%, 75%, 60%, 30%, 45%, and 70%, corresponding to TPU-6, TPU-8, TPU-10, TPU-12, TPU-14, and TPU-16. The TPU-10 not only have a filtration efficiency of 99.654% and the transmission rate is as high as 60%. Figure 7b shows the photograph of the TPU-10 nanofiber membrane with 60% transmittance. For air filters with a transmission of more than 50%, sufficient light can be transmitted through the room to meet indoor lighting requirements.

**Fig. 8:**
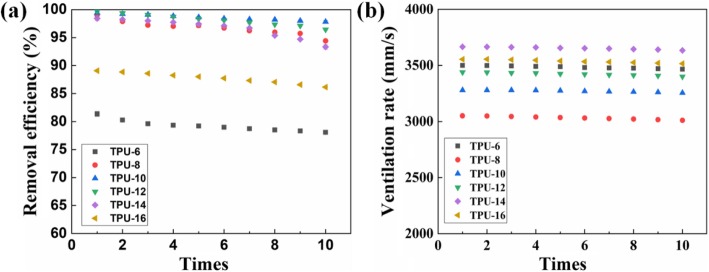
Reproducibility of the ventilation rate and removal efficiency of the composite fiber membrane. **a** Reproducibility of the removal efficiency. **b** Reproducibility of the ventilation rate

Considering that long-term filtration performance and high air flow are important factors in air filters, we have recycled TPU fiber membranes and continued to test filtration efficiency and ventilation rate, and the results are shown in Fig. 8. Figure 8a shows error bars for combined removal efficiency of 10 cycles of testing of PM2.5 filtration of TPU nanofiber membrane. After 10 passes of TPU-10 filtration, the filtration efficiency was only reduced by 1.6% (from 99.4 to 97.8%). In addition, an error bars for the aeration rates of the 10 test cycles for different TPU concentration fiber membranes are shown in Fig. 8b. The ventilation rate changed slowly and did not decrease significantly. After ten breath tests, the ventilation rate was only reduced by about 10 mm/s, indicating that the ventilation effect is very stable.

### Conclusion

In summary, we use a rotating bead spinneret for electrospinning to create a transparent air filter that can be produced in a large scale. By changing the concentration of TPU polymer in solution, not only significant PM2.5 removal efficiency (99.654%) is achieved, but also good optical transparency (60%) and ventilation rate (3480 mm/s) are achieved. In addition, by performing 10 cycles of filtration and gas venting tests on the TPU transparent air filter, the results showed that the filtration efficiency was only reduced by 1.6%, and the ventilation rate was changed very slowly and remained substantially unchanged. These results indicate that TPU nanofiber membranes prepared by electrospinning have many advantages such as good water repellency, good optical transparency, high ventilation rate, and high filtration performance, which can be used as filter materials in a lot of fields.

## Data Availability

All data generated or analyzed during this study are included in this published article.
